# New Factors Controlling Parent-Specific Genetic Control

**DOI:** 10.1371/journal.pbio.0040398

**Published:** 2006-10-17

**Authors:** Jason Underwood

In humans, two linear meters of DNA must miraculously compact down and fit into each cell’s nucleus. Special proteins known as histones act as the spools around which DNA is coiled and contorted. This system keeps the genome restricted to a reasonable space and also allows for dynamic changes in gene regulation. Different regions of the DNA can become decondensed and activated in accordance with developmental timing, cell type, or in response to the environment. Some regions of the genome remain silent for the life of the organism, while others must respond at the flip of a switch, turning particular genes on or off in response to cellular cues.

Humans and other animals have diploid genomes, meaning that they have two versions of each gene, one from each parent. These two copies, or alleles, can be regulated in concert or independent from one another. Genetic imprinting is a special case where gene expression is restricted to just one of the parental alleles. One interesting and well-studied example of imprinting occurs in a region of the genome where the neighboring genes Igf2 and H19 reside. The gene for Igf2, an insulin-like growth factor, is only expressed from the paternal allele, while the noncoding RNA gene, H19, is only expressed from the maternal allele. A small DNA region in between the two genes, appropriately called the imprinting control region (ICR), assigns the neighboring gene’s activity. The paternal allele ICR has small chemical modifications on the DNA known as methylation, and this is key to proper Igf2/H19 regulation. The mechanism by which only the paternal allele gets these modifications has long remained a mystery, but now a recent study indicates a link between a testis-specific protein and the paternal methylation of the ICR. The study by Petar Jelinic, Jean-Christophe Stehle, and Phillip Shaw demonstrates that in mice, this testis-specific factor, CTCFL, binds to the ICR and recruits other factors and enzymes that direct the methylation of this region.

The factor of interest, CTCFL, was discovered several years ago and became an interesting candidate for regulation of the Igf2/H19 region not only because of its testis expression pattern, but also because its amino acid sequence resembles another known DNA-binding protein, CTCF. This protein was known to bind to specific DNA sequences present in the ICR. As expected, the testis protein, CTCFL, could also bind to the same sequences. Then, the CTCFL protein was used as bait in a genetic fishing expedition to catch proteins that might physically interact with CTCFL. Interestingly, the two “fish” that were caught were both factors that are known to play key roles in gene regulation. One was a testis-specific component of the DNA-spooling complexes, a histone H2A protein variant. The other protein was an enzyme that can add methyl groups to other proteins. This enzyme, protein arginine methyltransferase 7 (PRMT7), was previously shown to add methyl groups to histone proteins, and these methyl modifications can have profound effects on the activity of the bound DNA region.

These new candidates for Igf2/H19 regulation were tested in a number of assays. After confirming that CTCFL proteins can physically bind the PRMT7 enzyme and histone proteins, the authors verified that they are expressed in the testis during the proper developmental stages to influence imprinting. Next, the authors used clues from previous studies on PRMT7, which indicated several candidate methyl targets of particular interest to gene regulation gurus, the histone proteins. Since CTCFL can bind to the enzyme and to histones, the authors postulated that perhaps CTCFL acts as a meeting place for the enzyme and the histones. Their observations were in agreement with this hypothesis, as they found that CTCFL significantly stimulated the efficiency with which PRMT7 could methylate the histone family members H2A and H4. But, could the activity of these factors and the methylation of the histones affect the methylation of the DNA itself, since it is this type of modification that tags the paternal allele? To address this question, the candidate genes as well as genes for the methyl transferring enzymes that work on DNA were injected in various combinations into frog eggs. A DNA sequence that resembles the mouse ICR region was also injected into the eggs, and a special assay was used to monitor the methylation of this foreign DNA. When all three key players—CTCFL, PRMT7, and the enzyme that methylates DNA, Dnmt3—were injected, significant DNA methylation was observed on the ICR sequence. Leaving out any of these components resulted in lower overall methylation. Taken together, it appears that these new components play important roles in paternal imprinting.

The authors propose a model to account for the methylation events on both histone proteins and on the ICR DNA. This study provides two new factors to tweak this fascinating genetic event at the Igf2/H19 region, but it is clear that this model will become even more complex in time. This region is arguably the best studied model for imprinting, and yet there’s so much left to be learned about these mysterious control mechanism guarding and prying at the genome.

